# Facile Preparation of a Cellulose-Based Thermoresponsive Gel for Rapid Water Harvesting from the Atmosphere

**DOI:** 10.3390/polym17162253

**Published:** 2025-08-20

**Authors:** Xiaoyu Wang, Hui Zhang, Xinxin Liu, Jie Du, Yingguang Xu

**Affiliations:** 1 School of Materials Science and Engineering, Hainan University, Haikou 570228, China; 23220856010055@hainanu.edu.cn (X.W.); lxx19910312@163.com (X.L.); dujie@hainanu.edu.cn (J.D.); 2 Shandong Key Laboratory of Preparation and Application of New Thermoplastic Elastomer Materials, Yantai 265702, China; 3 Hainan Academy of Inspection and Testing, Chengmai 571925, China

**Keywords:** atmospheric water harvesting, hydrogel, thermoresponsive, cellulose nanofibrils, lithium chloride

## Abstract

Atmospheric water harvesting, as an emerging water collection technology, is expected to mitigate water resource crises. Adsorption-based atmospheric water harvesting technology offers distinct advantages, including geographical independence and reduced reliance on ambient humidity levels. Herein, a thermoresponsive gel (PNIPAM/TO-CNF) integrated with lithium chloride was constructed to achieve accelerated moisture sorption and rapid desorption capabilities. In the designated PNIPAM/TO-CNF/LiCl gel, PNIPAM provided a temperature-responsive hydrophilic–hydrophobic transition network; the hydrophilicity and structural strength were enhanced by TO-CNF, the moisture absorption capacity was dramatically elevated by hygroscopic salt LiCl, and pore-forming agent polyethylene glycol created a favorable porous structure. This synergistic design endows the gel with an optimized hydrophilic network, temperature-responsive behavior, and a porous architecture conducive to water vapor transportation, thereby achieving rapid moisture absorption and desorption. Under 60% relative humidity, the gel exhibited a water vapor adsorption capacity of 144% within 1 h, reaching its maximum absorption capacity of 178% after 140 min. The gel exhibited an even more superior desorption performance: when heated to 70 °C, its moisture content rapidly decreased to 16% of its initial weight within 1 h, corresponding to the desorption of 91% of the total absorbed water. A simplified pore-forming methodology that enables the integration of temperature-responsive properties with efficient moisture transfer channels was reported in this paper, providing a viable design pathway for achieving accelerated adsorption–desorption cycles in atmospheric water harvesting.

## 1. Introduction

The global water scarcity crisis is increasingly severe, with populations in numerous cities worldwide facing water shortages [1]. Population growth, urbanization, and economic and social development are projected to increase urban industrial and domestic water demand by 50–80% over the next three decades [2,3]. The global urban population suffering from water scarcity is anticipated to rise from 933 million (one-third of the global urban population) in 2016 to between 1.693 billion and 2.373 billion (one-third to nearly half of the global urban population) by 2050. India is projected to be the most severely affected, with its urban water-scarce population increasing by 153 million to 422 million people [4]. Concurrently, water pollution in over 2000 sub-basins globally exacerbates water scarcity. The number of sub-basins experiencing water scarcity due to projected future nitrogen pollution is expected to triple. This deterioration implies that, by 2050, an additional 40 million square kilometers of basin area and three billion more people could face water scarcity [5]. Consequently, atmospheric water, estimated at over 10^13^ m^3^, is becoming critically significant as an alternative, renewable freshwater source. It can be harvested to mitigate current water scarcity [6], offering advantages including minimal pollution and continuous replenishment via the hydrological cycle, with limited geographical constraints. Atmospheric water harvesting (AWH) technology represents an emerging field developed to collect atmospheric moisture, contributing to efforts to alleviate water resource scarcity.

Currently, atmospheric water harvesting technologies are categorized into three main types: fog collection, dew collection, and sorption-based atmospheric water harvesting (SAWH) [7]. However, fog collection requires relatively high humidity levels [8,9], while dew collection also demands certain humidity conditions; moreover, lower relative humidity and higher temperatures significantly increase its energy consumption [10]. Lower environmental humidity (i.e., reduced total moisture content) yields a depressed dew point temperature. To initiate condensation, the actual temperature of the condensing surface must be lower than this dew point. Consequently, achieving surface temperatures sufficiently low to reach or surpass such depressed dew points under arid conditions necessitates enhanced refrigeration capacities or reduced heat sink temperatures, thus, in turn, elevating electrical energy consumption. Consequently, these methods face greater limitations compared to sorption-based atmospheric water harvesting. SAWH technology primarily employs adsorbents to capture atmospheric moisture. Subsequently, heating enables the release of trapped liquid water as vapor from the adsorbent, which is then condensed into liquid water via a collection device. Current adsorbents for SAWH include silica gel [11], zeolites [12], hygroscopic salts [13,14,15], metal–organic frameworks (MOFs) [16,17,18], and hygroscopic hydrogels [19,20,21]. Unlike other adsorbents, conventional hydrogels exhibit a relatively limited intrinsic sorption capacity in ambient air without the integration of supplemental hygroscopic agents. However, their unique polymer network structure provides internal water transport channels. When combined with other hygroscopic materials, they enable significantly faster moisture capture rates and enhanced water retention capabilities compared to conventional adsorbents. Beyond structural properties, the chemical diversity inherent to hydrogels enables multifunctionality, exemplified by stimuli-responsive behaviors such as thermoresponsiveness. As the most widely employed thermoresponsive hydrogel [22,23], poly(N-isopropylacrylamide) (PNIPAM) finds extensive applications in atmospheric water harvesting [20,24,25,26]. Below its lower critical solution temperature (LCST), PNIPAM maintains a hydrophilic state, facilitating adsorption of water molecules from air. Upon heating above the LCST, the hydrogel transitions to a hydrophobic state, enabling the rapid release of liquid water in vapor form. Furthermore, integration with functional materials imparts additional capabilities. For instance, coupling with photothermal conversion materials (e.g., carbon nanotubes, polypyrrole chloride, and graphene oxide) permits the conversion of solar energy into thermal energy, thereby elevating the hydrogel temperature above the desorption threshold [21,27].

Cellulose, the most abundant natural polymer on earth, frequently serves as a hydrogel precursor material [28,29,30,31]. Its numerous surface hydroxyl groups impart hydrophilicity, promoting moisture adsorption and rendering cellulose-based hydrogels suitable for atmospheric water harvesting applications [32,33,34,35,36]. Although hydrogels offer advantages, including network skeletons, chemical diversity, and high design flexibility, creating boundless potential and promising prospects for atmospheric water harvesting, current hydrogel-based atmospheric water harvesters still exhibit drawbacks. For example, certain materials achieve a high moisture absorption capacity but require prolonged durations, rendering them impractical for real-world production requirements [20,21]. Others enable rapid adsorption coupled with high moisture absorption but incur extended desorption times; shortening desorption necessitates excessively high heating temperatures, yet the desorption duration remains substantial regardless [37,38]. Tempo-oxidized cellulose nanofibrils (TO-CNF), as a cellulose derivative, exhibit extensive functional applications. Compared to CNF, TO-CNF has more carboxyl groups on its surface, which makes TO-CNF highly negatively charged and enables it to spontaneously and stably disperse. Therefore, a PNIPAM/TO-CNF/LiCl gel was developed to enhance adsorption kinetics via a porous structure fabricated using a porogen and freeze-drying. Integrating TO-CNF improves inherent hydrophilicity and, ultimately, the moisture absorption capacity, while leveraging PNIPAM thermoresponsiveness enables rapid desorption. This simultaneously achieves rapid moisture adsorption, a high moisture absorption capacity, and fast desorption attainable under low-heating-temperature conditions.

In this study, a PNIPAM/TO-CNF/LiCl gel was developed, leveraging abundant hydroxyl and carboxyl groups on TO-CNF surfaces to enhance adsorption kinetics, hydrophilicity, and the moisture absorption capacity. Through a facile pore-forming technique, hierarchical vapor transport channels were engineered within the hydrogel matrix, thereby achieving the simultaneous acceleration of both moisture adsorption and desorption processes. PNIPAM was utilized as the polymeric network backbone, which enables a thermally driven hydrophilic–hydrophobic transition upon heating. The hydrogel’s hydrophilicity and structural integrity were reinforced by TO-CNF (with its abundant hydroxyl and carboxylic groups) when integrated with PNIPAM. As a nanocellulosic material, cellulose nanofibrils (CNF) are characterized by a three-dimensional entangled network formed through random entanglement of highly flexible nanofibrils [39]. These nanofibrils typically exhibit higher aspect ratios and superior flexibility compared to cellulose nanocrystals (CNW) [40]. TO-CNF is synthesized through the selective oxidation of the primary C6 hydroxyl groups in cellulose nanofibrils to sodium carboxylate groups (−COO^−^Na^+^) within a TEMPO-mediated reaction system [41]. This modification imparts a substantial negative surface charge density to TO-CNF. Consequently, the incorporation of polar TO-CNF enhances the hydrophilicity of poly(N-isopropylacrylamide) (PNIPAm) matrices and elevates their water absorption capacity. Lithium chloride is employed as the primary hygroscopic agent, while polyethylene glycol (PEG) is utilized as a porogen to generate a well-defined porous architecture. This synergistic design confers the gel with an exceptional hydrophilic network, optimized vapor-permeable pores, and thermoresponsive properties, which collectively facilitate swift moisture adsorption–desorption cycles. At 30% and 60% relative humidity (RH), the gel achieved moisture uptakes of 60% and 144% of its dry weight within one hour, respectively, reaching peak adsorption capacities of 93% and 178% after 140 min. Its desorption performance proved even more remarkable: under heating at 70 °C, the gel reduced its moisture content to 16% in one hour, releasing 91% of the absorbed water. This efficiency stems from the synergistic action of PNIPAM’s hydrophilic–hydrophobic transition and the highly interconnected pore structure. Combining these advantages, the PNIPAM/TO-CNF/LiCl gel presents a promising, straightforward pore-forming solution to enhance atmospheric water harvesting technologies.

## 2. Materials and Methods

### 2.1. Materials

N-isopropyl acrylamide (NIPAm, 98%), ammonium persulfate (APS, 98%), N,N’-methylenebisacrylamide (MBA, 99%), N,N,N,N-Tetramethylethylenediamine (TEMED, 99%), lithium chloride (LiCl, 98%), and poly (ethylene glycol) (average Mn: ~8000) were purchased from Shanghai Macklin Biochemical Co., Ltd. (Shanghai, China). TEMPO-oxidized cellulose nanofibers (TO-CNF) were purchased from Shanghai ScienceK Nanotechnology Co., Ltd. (Shanghai, China). The TO-CNF exhibited a carboxyl content of 1.2–3.0 mmol/g, with diameters of 5–20 nm and lengths of 1–3 μm. All materials were used without further purification.

### 2.2. Fabrication of PNIPAM/TO-CNF/LiCl Gel

The PNIPAM/TO-CNF/LiCl gel was synthesized via free-radical polymerization. Specifically, 0.5 g of N-isopropylacrylamide (NIPAM) and 0.015 g of N,N′-methylenebisacrylamide were dissolved in 10 mL of deionized water within a beaker under constant stirring [42]. Subsequently, 0–8 g of 1 wt% TEMPO-oxidized cellulose nanofibers (TO-CNF) and 0–1.5 g of polyethylene glycol (PEG) were added to the mixture and homogenized. In a separate beaker containing 10 mL of deionized water, 0.37 g of ammonium persulfate (APS) was dissolved. Approximately 1 mL of this APS solution was introduced dropwise into the NIPAM/TO-CNF suspension, followed by the addition of 20 μL of N,N,N′,N′-tetramethylethylenediamine (TEMED). Following thorough mixing, the precursor solution was transferred into a polytetrafluoroethylene (PTFE) mold. Polymerization proceeded undisturbed for 24 h, yielding 11.5–20 g of PNIPAM/TO-CNF gel. The PNIPAM/TO-CNF gel was removed from the mold and immersed in deionized water for 1-2 days to leach unreacted monomers, with the water being replenished at regular intervals every few hours. Afterwards, the gel underwent freeze-drying. It was then saturated by immersion in LiCl solutions of varying concentrations for 24 h and subjected to a final freeze-drying step, yielding the dry PNIPAM/TO-CNF/LiCl composite gel. Following immersion in a lithium chloride (LiCl) solution and freeze-drying, the dry PNIPAM/TO-CNF/LiCl gel yielded approximately 0.8–1.5 g. The PNIPAM/TO-CNF/LiCl gel was maintained in its bulk form without any processing.

### 2.3. Characterizations

Following sample preparation, specimens were immersed in deionized water until reaching a swelling equilibrium. Subsequently, they were subjected to freeze-drying for 48 h. After immersion in lithium chloride solution for 24 h, a secondary freeze-drying cycle was performed for 48 h. A 15 nm-thick gold conductive layer was deposited via ion sputtering prior to morphological characterization. Fracture surface topography was examined using field emission scanning electron microscopy (SEM, Verios G4 UC, Thermo scientific, Waltham, MA, USA) at an acceleration voltage of 15 kV under high vacuum conditions. Energy-dispersive X-ray spectroscopy (EDS) was further utilized for elemental distribution analysis across fracture surfaces through an area-scanning mode. Samples were analyzed via Attenuated Total Reflection Fourier Transform Infrared Spectroscopy (ATR-FTIR) using a Fourier-transform infrared spectrometer (FTIR-650S, TIANJIN GANGDONG SCI. & TECH. Co., Ltd., Tianjin, China). Prior to testing, specimens were freeze-dried, and surfaces were leveled. Spectra were acquired in the wavenumber range of 4000 cm^−1^ to 650 cm^−1^ for TO-CNF, PNIPAM, and PNIPAM/TO-CNF/LiCl gel, respectively. The phase transition behavior of the hydrogel was characterized by differential scanning calorimetry (DSC 250, TA Instruments, Newcastle, WA, USA) using standard aluminum crucibles, with measurements conducted under a nitrogen atmosphere at a heating rate of 2 °C min^−1^. XRD profiles were obtained by an X-ray Diffractometer (XRD, Bruker D8 Venture, Bruker Corporation, Billerica, MA, USA). Prior to X-ray diffraction analysis, the lyophilized samples were sectioned to ensure planar surfaces. Diffraction patterns were acquired over a 2θ range of 10–80°, employing a scanning rate of 10° min^−1^. Collected water samples were filtered through a 0.45 μm membrane before analyzing ionic concentrations via inductively coupled plasma optical emission spectrometry (ICP-OES, Agilent 720ES, Agilent Technologies, Santa Clara, CA, USA) to evaluate water quality compliance with predetermined standards. The TGA curves were obtained using a thermogravimetric analyzer (TGA, TG 209 F3, NETZSCH, Selb, Germany) under a nitrogen atmosphere with a heating rate of 10 °C/min.

### 2.4. Hygroscopicity and Desorption Testing of Gel

Moisture Adsorption Test: PNIPAM/TO-CNF/LiCl gel was promptly transferred to sealed bags after freeze-drying. Saturated salt solutions (sodium bromide for 60% RH; magnesium chloride for 30% RH) were employed to humidify the injected air and maintain the entire sealed environment at constant relative humidities of 30% or 60% RH. Temperature and humidity within the chamber were continuously monitored with a hygrometer. Samples were retrieved at scheduled intervals and positioned on an analytical balance for mass measurement. Moisture uptake was defined as the percentage of adsorbed water relative to the pre-adsorption mass, expressed by Equation (1):

Mass change = (W_1_ − W_d_/W_d_) × 100% (1)

W_1_ represents the sample mass measured at a designated relative humidity and time point, while W_d_ denotes the lyophilized post-test sample mass.

Desorption Test: After reaching moisture-saturated states under 30% or 60% RH, the gel was subjected to desorption in forced-air convection ovens at specified temperatures. Samples were retrieved at scheduled intervals and positioned on an analytical balance for mass measurement. The desorption ratio was defined as the percentage of desorbed moisture relative to adsorption-equilibrated moisture content, expressed by Equation (2):

Desorption ratio = (W_e_ − W_2_)/(W_e_ − W_d_) × 100% (2)

W_e_ is the sample mass at adsorption equilibrium, W_2_ is the sample mass following a period of drying, and W_d_ is the freeze-dried mass of the test specimen.

Field-Mimetic Adsorption–Desorption Cycling Test: Rapid moisture capture was performed within an open-configuration test apparatus. Upon reaching saturated adsorption, the system was sealed, and desorption was initiated using a polyimide heater. A cooling fan that enabled the temperature of the upper inclined surface to remain lower than that of other places was applied in the test, resulting in water vapor condensation into droplets and a subsequent downward flow into the collection tank. During testing, the ambient temperature was maintained at 23–27 °C, with a relative humidity of 50% to 80%, while the heating temperature of the polyimide heater was adjusted to 60–80 °C, according to the experimental protocol. In the adsorption/desorption experiments, the gel samples were tested as independent gel forms.

## 3. Results and Discussion

### 3.1. Characterization of PNIPAM/TO-CNF/LiCl Gel

**Figure 1:**
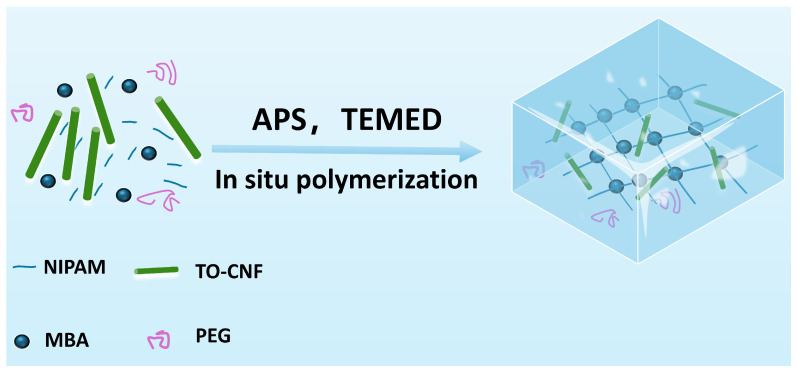
Schematic illustration of the polymerization of PNIPAM/TO-CNF/LiCl hydrogel.

In this study, PNIPAM/TO-CNF/LiCl gel was fabricated via free radical polymerization. Figure 1 illustrates the schematic diagram of the polymerization process of PNIPAM/TO-CNF/LiCl hydrogels. NIPAM, MBA, and TO-CNF all exhibited excellent water solubility. After the complete dissolution of the monomers in water, upon the introduction of APS and TEMED, persulfate ions (S_2_O_8_^2−^) underwent a reduction facilitated by TEMED. This initiated a homolytic cleavage reaction, generating sulfate radical anions (SO_4_^−^). TEMED functioned as a reducing agent, catalyzing the decomposition of APS into radicals and enabling the reaction to proceed rapidly at an ambient temperature. Subsequently, the sulfate radical anions (SO_4_^−^) attacked the vinyl groups of the monomers (NIPAM and MBA), generating the initial monomer radicals. These monomer radicals underwent continuous chain propagation by adding to the C=C bonds of other monomers or the cross-linker, ultimately forming the three-dimensional network structure of PNIPAM. Throughout this polymerization process, the amide groups (-C=O and -N-H) on the PNIPAM chains formed hydrogen bonds with the hydroxyl groups (-OH) and carboxyl/carboxylate groups (-COOH/-COO^−^) present on the TO-CNF surface. Furthermore, TO-CNF, being rigid nanoscale cellulose fibers characterized by a high aspect ratio (length significantly exceeding diameter), became physically entangled throughout the macropores of the developing PNIAPM network. Consequently, the effective physical interactions between PNIPAM and TO-CNF led to the formation of a semi-interpenetrating polymer network (semi-IPN) structure during the in situ polymerization process. During the polymerization process, PEG exhibited no reactivity with either PNIPAM or TO-CNF. Consequently, PEG became physically entrapped within the PNIPAM network, thereby creating a porous architecture. The synthesized PNIPAM/TO-CNF gel was immersed in deionized water to remove unreacted monomers and PEG, followed by freeze drying to obtain the dehydrated PNIPAM/TO-CNF gel. This dried gel was subsequently soaked in an LiCl solution to facilitate LiCl loading into the PNIPAM/TO-CNF matrix. A final freeze-drying step yielded the PNIPAM/TO-CNF/LiCl composite gel.

**Figure 2:**
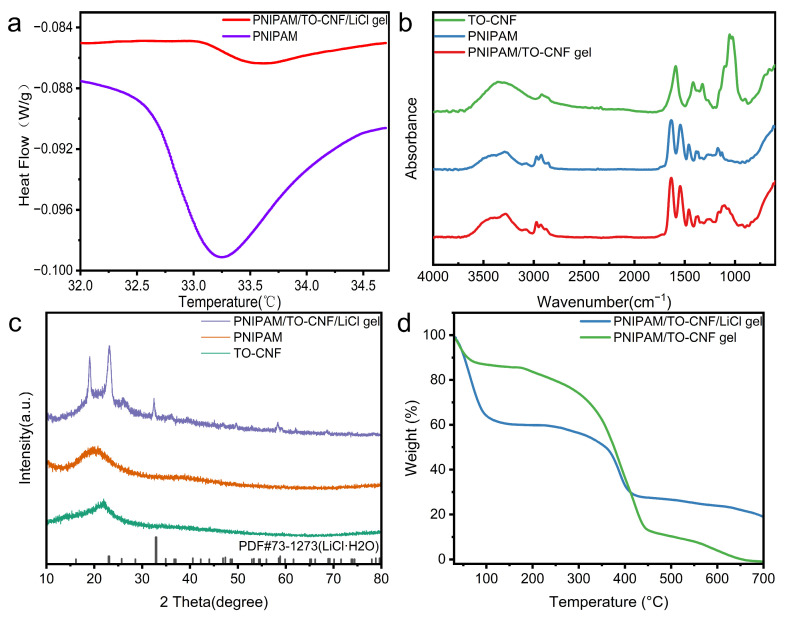
(**a**) DSC thermograms of PNIPAM/TO-CNF/LiCl gel and PNIPAM. (**b**) FTIR spectra of PNIPAM/TO-CNF/LiCl gel, TO-CNF, and PNIPAM. (**c**) XRD patterns of PNIPAM/TO-CNF/LiCl gel, TO-CNF, and PNIPAM. (**d**) TGA curves of PNIPAM/TO-CNF/LiCl gel and PNIPAM/TO-CNF gel.

The primary framework consisted of thermosensitive PNIPAM, which exhibited distinct hydrophilic properties below its lower critical solution temperature (LCST) but transitioned to hydrophobic characteristics when heated above the LCST. When the temperature rose above the LCST of the gel, the hydrogen bonds between the amide groups (-C=O and -N-H) on the PNIPAM chains and the surrounding water molecules began to destabilize and break. Concurrently, the hydrophobic isopropyl side chains (-CH(CH_3_)_2_) experienced an intensified thermal motion. This increase in thermal energy significantly enhanced intermolecular hydrophobic interactions. Consequently, the previously structured, ordered hydration shells surrounding the hydrophilic polymer chains underwent a breakdown. Collectively, these changes were expected to promote a transition of liquid water trapped within the gel network into water vapor during desorption. This process was anticipated to facilitate the rapid desorption kinetics targeted in our design. Differential scanning calorimetry (DSC) was employed to determine the LCST of the thermosensitive gel. As illustrated in Figure 2a, the pure PNIPAM gel exhibited an LCST of 33.24 °C, while the PNIPAM/TO-CNF/LiCl composite gel demonstrated a slightly elevated LCST of 33.65 °C. The elevation of the LCST confirmed the effective integration of TO-CNF within PNIPAM and signified that incorporating TO-CNF—abundant in hydroxyl and carboxyl groups—enhanced the gel’s hydrophilicity. This increased hydrophilicity accelerated moisture adsorption kinetics and elevated moisture sorption capacity during atmospheric water harvesting. The elevated LCST further enabled the gel to maintain its hydrophilic state during high-temperature daytime periods, thereby accelerating moisture capture kinetics and achieving continuous sorption throughout diurnal cycles. Figure 2b presents FT-IR spectra of TO-CNF, PNIPAM, and PNIPAM/TO-CNF/LiCl gels. For TO-CNF, which is a polymeric compound composed of glucose units with abundant surface hydroxyl groups, the broad peak at 3361 cm^−1^ was attributed to O-H stretching vibrations. The absorption peak at 2919 cm^−1^ corresponded to C-H stretching vibrations of cellulose, while the peak at 1590 cm^−1^ related to stretching vibrations of carboxylate groups. An absorption peak at 1051 cm^−1^ was observed, assigned to the C-O-C stretching vibration. All the characteristic peaks collectively agreed with the structural features of TO-CNF. Regarding PNIPAM, the overlapping double peaks at 3422 and 3291 cm^−1^ were attributed to asymmetric and symmetric N-H stretching vibrations, respectively. The infrared spectrum displayed characteristic absorption peaks at 2975 cm^−1^ (C–H stretching vibration), 2925 cm^−1^ (≡C–H stretching vibration), 2852 cm^−1^ (C–H stretching vibration), and 1459 cm^−1^ (asymmetric bending of methyl groups), respectively. Characteristic absorptions of amide I and amide II bands appeared at 1631 and 1546 cm^−1^. The FT-IR spectrum of PNIPAM/TO-CNF/LiCl gel largely resembled that of pure PNIPAM but exhibited an additional absorption peak at 1105 cm^−1^, which was characteristic of the β-(1,4)-glycosidic linkage of cellulose ether. The peaks at 3422 cm^−1^ and 3291 cm^−1^ exhibited notably enhanced intensities, attributable to the concurrent stretching vibrations of N–H and O–H functional groups. The FTIR spectrum verified the homogeneous integration of PNIPAM and TO-CNF, confirming the successful fabrication of the composite gel.

Figure 2c displays XRD patterns of TO-CNF, PNIPAM, and PNIPAM/TO-CNF/LiCl gel for crystalline phase analysis, enabling material identification and structural comparison. PNIPAM exhibited a broad amorphous diffraction band around 20°. TO-CNF showed a characteristic crystalline peak at 22°, corresponding to the (200) crystallographic plane of cellulose Iβ. The relatively low crystallinity index of the commercially sourced TO-CNF material resulted in a less pronounced crystalline peak. The amorphous region of TO-CNF exhibited a broad diffuse peak at 18–20°. In the XRD pattern of PNIPAM/TO-CNF/LiCl gel, a broad band indicative of amorphous PNIPAM appeared near 20°, consistent with pure PNIPAM. Distinct from pure PNIPAM, the PNIPAM/TO-CNF/LiCl gel exhibited two sharp diffraction peaks at 19° and 23°. These peaks corresponded to characteristic PEG diffraction, belonging to the (120) and (112) crystallographic planes of PEG, respectively [43]. Additionally, minor sharp peaks at 32°, 49°, and 58° indicated the presence of lithium chloride monohydrate (LiCl·H_2_O), confirming the successful incorporation of lithium salts primarily existing in a free ionic state. Notably, no anhydrous LiCl characteristic peaks were observed in the PNIPAM/TO-CNF/LiCl pattern, which were attributed to the conversion of anhydrous LiCl to its monohydrate form during prolonged air exposure in sample preparation and characterization. Prolonged atmospheric exposure induces the transformation of lithium chloride monohydrate crystallites into an anhydrous lithium chloride aqueous solution within the hydrogel due to its high hygroscopicity, thereby resulting in the vanishing of characteristic lithium chloride monohydrate diffraction peaks in XRD patterns. Analysis of the XRD diffractograms confirms the successful incorporation of lithium chloride (LiCl) into the gel structure.

Figure 2d displays the TGA curves of PNIPAM/TO-CNF/LiCl gel and PNIPAM/TO-CNF gel. For PNIPAM/TO-CNF gel, rapid weight loss occurred below 100 °C due to the fast evaporation of adsorbed water vapor. Although the PNIPAM/TO-CNF gel contained no loaded LiCl, its high hydrophilicity enabled significant water vapor adsorption. Between 175 °C and 450 °C, rapid weight loss was observed, corresponding to the decomposition of PNIPAM and TO-CNF components within the PNIPAM/TO-CNF gel; the decomposition rate increased with the temperature until complete degradation. For the PNIPAM/TO-CNF/LiCl gel, rapid weight loss below 100 °C was also attributed to adsorbed water evaporation, with a greater magnitude than the PNIPAM/TO-CNF gel. This resulted from the substantial water vapor adsorption capacity imparted by the loaded LiCl. A second rapid weight loss stage occurred between 220 °C and 440 °C, similarly arising from the decomposition of PNIPAM and TO-CNF. The incorporation of LiCl elevated the onset temperature of this second stage to 220 °C, suggesting enhanced thermal stability. Finally, the PNIPAM/TO-CNF/LiCl gel retained 19% residual weight, corresponding to the contained LiCl. Finally, TGA results demonstrate that PNIPAM/TO-CNF/LiCl gel exhibited notable thermal stability, thus holding potential for applications in atmospheric water harvesting.

**Figure 3:**
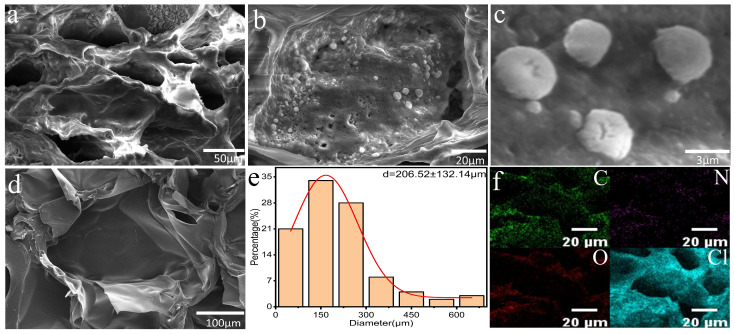
(**a**–**c**) SEM micrographs of PNIPAM/TO-CNF/LiCl gel. (**d**) SEM micrograph of PNIPAM/TO-CNF gel. (**e**) Pore Size Distribution of PNIPAM/TO-CNF/LiCl gel. (**f**) EDS elemental mapping of the PNIPAM/TO-CNF/LiCl gel.

As evidenced in Figure 3a, the PNIPAM/TO-CNF/LiCl gel demonstrated favorable porosity. This microstructure arose from the dual effects of lyophilization and the porogenic agent PEG, which collectively facilitated enhanced water vapor transportation. During PNIPAM cross-linking, the non-reactive PEG becomes entrapped within the PNIPAM network. Subsequent removal of PEG via prolonged aqueous immersion generated cavity structures in the polymeric skeleton, thereby establishing favorable porosity within the hydrogel. As clearly observed from Figure 3b,c, the pore surfaces of the PNIPAM/TO-CNF/LiCl gel were rough and exhibited spherical white particles. In contrast, Figure 3d demonstrates that the pore surfaces of the PNIPAM/TO-CNF gel without LiCl immersion remained smooth. It can, therefore, be concluded that the deposits on the pore surfaces of the PNIPAM/TO-CNF/LiCl gel consisted of LiCl. Figure 3e presents the pore size distribution of the PNIPAM/TO-CNF/LiCl gel. The majority of pores fell within the 50–300 μm range, with an average diameter of 206.52 μm. This microporous structure facilitated enhanced water vapor transportation into the gel network during adsorption and enabled rapid vapor release during desorption, thereby providing an optimal structural foundation for atmospheric water harvesting applications. Figure 3f reveals a uniformly distributed layer of lithium chloride coating the skeleton surface, evidenced by the homogeneous elemental distribution of chlorine across the gel matrix. This morphology indicates that the simple post-soaking technique effectively achieved uniform dispersion of lithium chloride throughout both the pores and skeleton framework of the gel. It can also be proved that the substance attached to the PNIPAM/TO-CNF/LiCl gel, as seen in Figure 3b,c was lithium chloride. Such homogeneous distribution facilitated the efficient adsorption of atmospheric water molecules, thereby enabling the PNIPAM/TO-CNF/LiCl gel to exhibit rapid moisture absorption kinetics.

### 3.2. The Atmospheric Water Harvesting Performance of PNIPAM/TO-CNF/LiCl Gel

The critical performance metrics for atmospheric water harvesting in PNIPAM/TO-CNF/LiCl gel encompass hygroscopic kinetics, equilibrium moisture uptake, and desorption rates. Hygroscopic kinetics were quantified by monitoring mass variations in the gel under constant humidity conditions. The moisture capture mechanism involved hygroscopic anhydrous lithium chloride salts immobilized on the gel scaffold. Upon air exposure, the anhydrous lithium chloride salts adsorbed water vapor to form LiCl·H_2_O, which progressively transitioned into an aqueous LiCl solution through vapor accumulation and further hydration. Throughout this process, adsorbed water molecules diffused into and were stored within the gel matrix. Moreover, the hydroxyl and carboxyl groups abundant on the TO-CNF surface conferred enhanced hydrophilicity to the porous gel skeleton upon TO-CNF incorporation, consequently improving water retention performance. Continuous vapor adsorption occurred when the partial pressure of ambient water vapor exceeded the saturated vapor pressure of the surface LiCl solution layer, and the process persisted until a vapor–liquid equilibrium was attained. The hygroscopic kinetics and ultimate moisture uptake capacity of PNIPAM/TO-CNF/LiCl gel were primarily governed by three factors: the mass fraction of TO-CNF, the concentration of anhydrous LiCl, and the mass of porogenic agent PEG.

**Figure 4:**
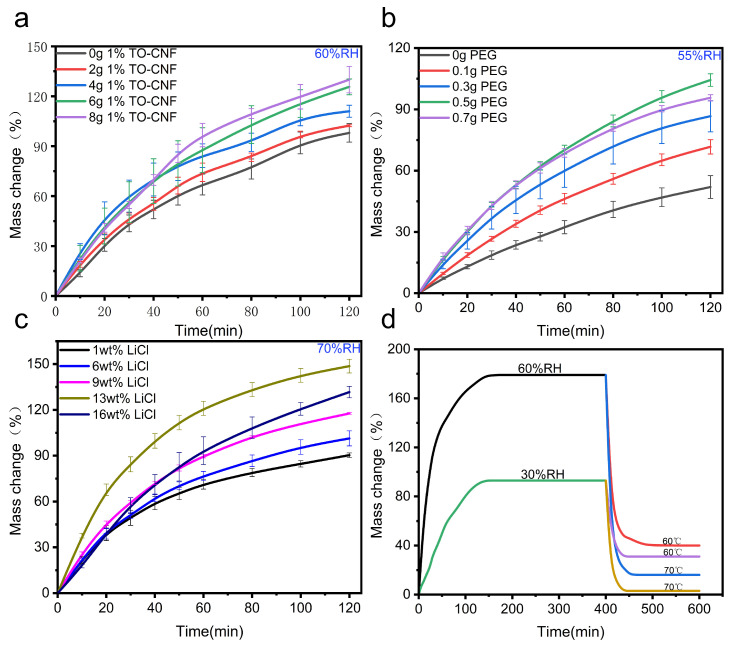
(**a**) Performance comparison of gels with varying TO-CNF content. (**b**) Performance comparison of gels at different PEG masses. (**c**) Performance comparison of gels with varying LiCl concentrations. (**d**) Optimum performance of the PNIPAM/TO-CNF/LiCl gels.

Figure 4a illustrates the optimization of TO-CNF loading, wherein the moisture uptake capacity increased progressively with the incorporation of 1 wt% TO-CNF. This enhancement stemmed from improved gel hydrophilicity. However, exceeding 8 g of 1 wt% TO-CNF resulted in system viscosity elevation that compromised successful gelation. Remarkably, the sample containing 8 g of 1 wt% TO-CNF achieved a 30% higher moisture capacity than its TO-CNF-free counterpart, confirming that heightened hydrophilicity facilitated enhanced water retention within the gel matrix. Pore-forming agent PEG was subsequently optimized. As illustrated in Figure 4b, the water absorption of the PNIPAM/TO-CNF/LiCl gel initially increased and subsequently decreased with the increasing PEG mass. Maximum absorption enhancement reached 50%, indicating that the porogen PEG effectively generated additional porous structures, thereby providing superior vapor transport channels and storage space for water molecules within the gel. However, a reduction in water absorption occurred when the PEG mass exceeded 0.5 g, likely attributable to structural weakening and a pore collapse resulting from excessive porosity formation. Critical optimization addressed LiCl loading (Figure 4c). As the key hygroscopic component, LiCl demonstrated the most pronounced impact, with capacity peaking at 60% enhancement. Excessive LiCl concentrations, however, primarily clogged the micropores within the gel network. This impaired vapor transport efficiency and substantially increased the gel’s dry mass, resulting in only marginal gains in adsorbed water molecules relative to the augmented mass. Following sequential optimization of these parameters, the gel attained an equilibrium moisture uptake of 0.93 g g^−1^ at 30% RH and 1.79 g g^−1^ at 60% RH. Saturation occurred within 140 min, with rapid kinetic performance achieving 80% saturation within 60 min under 60% RH. Meanwhile, the PNIPAM/TO-CNF/LiCl gel also demonstrated exceptional desorption performance (Figure 4d): dehydration efficiency reached 67% (30% RH) and 75% (60% RH) after 60 min at 60 °C, accelerating further to 96% (30% RH) and 91% (60% RH) at 70 °C. These results highlight synergistic thermoresponsiveness from PNIPAM and optimized water transport pathways, enabling rapid liquid-to-vapor phase conversion during thermal regeneration. Table 1 confirms that the PNIPAM/TO-CNF/LiCl gel demonstrated superior sorption/desorption kinetics with lower desorption temperature requirements.

**Figure 5:**
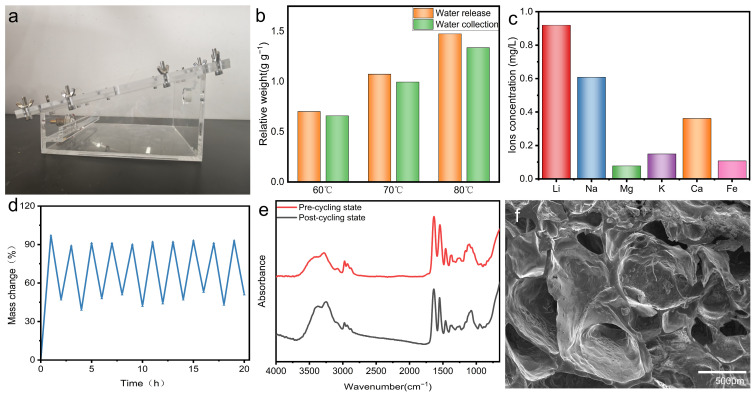
(**a**) Photograph of the atmospheric water harvesting (AWH) device. (**b**) Water collection performance of the PNIPAM/TO-CNF/LiCl gel within the device. (**c**) Quality of the collected water. (**d**) Cyclability evaluation of the PNIPAM/TO-CNF/LiCl gel. (**e**) FTIR spectral comparison of PNIPAM/TO-CNF/LiCl gel pre- and post-cycling. (**f**) SEM imaging of PNIPAM/TO-CNF/LiCl gel post-cycling.

A water harvesting device was constructed to validate the atmospheric water harvesting capability of PNIPAM/TO-CNF/LiCl gel. The apparatus featured a 30–inclined top plate with a cooling system to maintain sub-ambient temperatures, preferentially condensing vapor on the plate rather than the sidewalls. Condensed water flowed along the inclined surface into a collection reservoir via gravity. Internally, thermostatic polyimide heaters facilitated gel regeneration. Figure 5b illustrates temperature-dependent desorption kinetics, showing increased water release from the gel and enhanced collection yields with rising temperatures. The system collected approximately 90% of released vapor across three temperature regimes, confirming efficient operational performance.

Water quality analysis (Figure 5c) recorded lithium ions at 0.92 mg L^−1^. While the WHO lacks defined lithium thresholds, prolonged exposure risks necessitate the consideration of post-collection filtration for long-term use. For sodium ions, the WHO guideline value is less than 50 mg/L [48]. The concentration of sodium ions in the water collected by the hydrogel is 0.61 mg/L, well below the WHO guideline. Furthermore, the WHO has not established health-based guideline values for calcium and magnesium ions. However, based on water hardness, it provides general guidance with threshold concentrations of 300 mg/L for calcium and 500 mg/L for magnesium. The concentrations of calcium and magnesium ions in the hydrogel-collected water (0.36 mg/L and 0.08 mg/L, respectively) are far below these thresholds. For potassium ions, the WHO does not provide a health-based guideline value, as the concentrations typically found in drinking water are well below levels of health concern. The WHO guidance level for iron is 0.3 mg/L. The iron ion concentration in the hydrogel-collected water is 0.11 mg/L, also lower than this guidance level. Therefore, based on this analysis, the water harvested from the atmosphere demonstrates good water quality. Cyclic stability testing subjected the gel to ten consecutive adsorption (60% RH, 1 h) and desorption (oven, 1 h) cycles. Figure 5d shows that both the adsorption and desorption capacities of the PNIPAM/TO-CNF/LiCl gel demonstrated minimal variations throughout ten adsorption–desorption cycles. This consistent performance clearly demonstrates the excellent stability of the PNIPAM/TO-CNF/LiCl gel. Figure 5e reveals no significant changes in the FTIR spectra of the PNIPAM/TO-CNF/LiCl gel after cycling compared to its pre-cycled counterpart. Correspondingly, Figure 5f demonstrates that SEM micrographs of the cycled PNIPAM/TO-CNF/LiCl gel revealed the preservation of its well-defined porous architecture. These results collectively confirm the exceptional structural integrity and cycling stability of the PNIPAM/TO-CNF/LiCl gel composite.

## 4. Conclusions

In conclusion, a PNIPAM/TO-CNF/LiCl gel was constructed as an advanced atmospheric water harvesting material, utilizing PNIPAM’s thermoresponsive hydrophilic/hydrophobic switching capability to accelerate desorption kinetics. The strategic incorporation of PEG as a porogen yielded optimized vapor-transport channels, enabling rapid moisture capture and exceptional cyclic stability. Building upon this innovation, the work strategically incorporated strongly hydrophilic TEMPO-oxidized cellulose nanofibers (TO-CNFs) into the PNIPAM/TO-CNF/LiCl gel system. This integration effectively constructed a networked hydrophilic architecture with abundant binding sites, significantly enhancing both the hydrophilicity and moisture sorption/storage capacity of the composite gel. Integrated into an atmospheric water harvester, the gel achieved 6–8 daily adsorption/desorption cycles, with peak water production of 10.4 L kg^−1^ per day. This work integrated thermoresponsive properties, the abundant hydroxyl and carboxylic groups in cellulose, and porogenic characteristics to achieve rapid moisture sorption–desorption cycles. It proposed an innovative atmospheric water harvesting approach and provided a novel solution for distributed water sources to address water scarcity challenges.

## Figures and Tables

**Table 1:** Comparative hygroscopic performance of atmospherically harvested water materials.

Name	Sorption Water Capacity (g g^−1^) [RH]	Adsorption Time [min]	Desorption Water Capacity (g g^−1^) [°C]	Desorption Time [min]	Ref.
Bina/FCNT	5.6 [70%]	660	5.10 [80 °C]	40	[21]
PAMPS-CNT-LiCl	1.87 [60%]	600	1.72 [90 °C]	200	[44]
CNF/LiCl scaffolds	1.15 [57%]	720	0.98 [60 °C]	120	[37]
PAM 75%sat LiCl	3.93 [70%]	600	2.91 [70 °C]	120	[38]
PDMAPS-LiCl	0.62 [30%]	120	0.5 [80 °C]	30	[45]
LiCl@rGO–SA	2.85 [60%]	480	2.73 [90 °C]	200	[46]
CAL gel	0.79 [30%]	120	0.51 [65 °C]	60	[47]
PNIPAM/TO-CNF/LiCl gel	0.93 [30%] 178 [60%]	140	0.62 [30%] [60 °C] 1.37 [60%] [60 °C]	80	This study

## Data Availability

Data is contained within the article (The original contributions presented in this study are included in the article. Further inquiries can be directed to the corresponding authors).
